# Synthesis and crystal structures of two racemic 2-heteroaryl-3-phenyl-2,3-di­hydro-4*H*-pyrido[3,2-*e*][1,3]thia­zin-4-ones

**DOI:** 10.1107/S2056989024005103

**Published:** 2024-06-04

**Authors:** Hemant P. Yennawar, Tapas K. Mal, Mark A. Olsen, Anthony F. Lagalante, Evelyn M. Louca, Aloura D. Gavalis, Lee J. Silverberg

**Affiliations:** a Department of Biochemistry and Molecular Biology Pennsylvania State University University Park PA 16802 USA; b Department of Chemistry The Pennsylvania State University University Park PA 16802 USA; c Mendel Science Center Villanova University, 800 Lancaster Avenue Villanova PA 19085 USA; d Pennsylvania State University, Schuylkill Campus, 200 University Drive, Schuylkill Haven, PA 17972, USA; University of Neuchâtel, Switzerland

**Keywords:** crystal structure, thia­zine, envolope pucker, pyridine, heteroar­yl, indole, thio­phene, water channel, C—H⋯N weak hydrogen bonds

## Abstract

3-Phenyl-2-(thio­phen-3-yl)-2,3-di­hydro-4*H*-pyrido[3,2-*e*][1,3]thia­zin-4-one (C_17_H_12_N_2_OS_2_, **1**) and 2-(1*H*-indol-3-yl)-3-phenyl-2,3-di­hydro-4*H*-pyrido[3,2-*e*][1,3]thia­zin-4-one 0.438-hydrate (C_21_H_15_N_3_OS·0.438H_2_O, **2**) crystallize in space groups *P*2_1_/*n* and *C*2/*c*, respectively. The asymmetric unit in each case is comprised of two parent mol­ecules, albeit of mixed chirality in the case of **1** and of similar chirality in **2** with the enanti­omers occupying the neighboring asymmetric units. Structure **2** also has water mol­ecules (partial occupancies) that form continuous channels along the *b***-**axis direction. The thia­zine rings in both structures exhibit an envelope conformation. Inter­molecular inter­actions in **1** are defined only by C—H⋯O and C—H⋯N hydrogen bonds between crystallographically independent mol­ecules. In **2**, hydrogen bonds of the type N—H⋯O between independent mol­ecules and C—H⋯N(π) type, and π–π stacking inter­actions between the pyridine rings of symmetry-related mol­ecules are observed.

## Abstract 2

3-Phenyl-2-(thio­phen-3-yl)-2,3-di­hydro-4*H*-pyrido[3,2-*e*][1,3]thia­zin-4-one (C_17_H_12_N_2_OS_2_, **1**) and 2-(1*H*-indol-3-yl)-3-phenyl-2,3-di­hydro-4*H*-pyrido[3,2-*e*][1,3]thia­zin-4-one 0.438-hydrate (C_21_H_15_N_3_OS·0.438H_2_O, **2**) crystallize in space groups *P*2_1_/*n* and *C*2/*c*, respectively. The asymmetric unit in each case is comprised of two parent mol­ecules, albeit of mixed chirality in the case of **1** and of similar chirality in **2** with the enanti­omers occupying the neighboring asymmetric units. Structure **2** also has water mol­ecules (partial occupancies) that form continous channels along the *b***-**axis direction.

## 1. Chemical context

The 2,3-disubstituted-2,3-di­hydro-4*H*-pyrido[3,2-*e*][1,3]thia­zin-4-one scaffold features a pyridine ring fused to a thia­zine ring at the 5 and 6 positions. Compounds with this scaffold have previously shown anti­bacterial (Nayak *et al.*, 2022 ▸) anti­cancer (Arya *et al.*, 2014 ▸; Wang *et al.*, 2015 ▸), glycosidase inhibitory (Li *et al.*, 2012 ▸), and anti­fungal bioactivity (Liporagi-Lopes *et al.*, 2020 ▸). A compound previously reported by us, 2,3-diphenyl-2,3-di­hydro-4*H*-pyrido[3,2-*e*][1,3]thia­zin-4-one (Silverberg *et al.*, 2015 ▸; Yennawar *et al.*, 2014 ▸), inhibited growth of two kinetoplastid parasites, *Trypanosoma brucei* and *Crithidia fasciculata* (Malfara *et al.*, 2021 ▸). The effect was especially inter­esting for *T. brucei*, which causes African Sleeping Sickness (Human African Trypanosomiasis, HAT). A series of 2-aryl-3-phenyl-2,3-di­hydro-4*H*-pyrido[3,2-*e*][1,3]thia­zin-4-ones was then synthesized, with various substituents on the C-aryl ring. Five of these compounds (*p*- and *m*-CF_3_, *p*- and *m*-Br, *p*-CH_3_) showed much stronger activity against *T. brucei* than 2,3-diphenyl-2,3-di­hydro-4*H*-pyrido[3,2-*e*][1,3]thia­zin-4-one (Silverberg *et al.*, 2021 ▸). A series of 3-aryl-2-phenyl-2,3-di­hydro-4*H*-pyrido[3,2-*e*][1,3]thia­zin-4-ones has since been synthesized with various substituents on the N-aryl ring and is currently undergoing testing.

Using our simple 2,4,6-tripropyl-1,3,5,2,4,6-trioxatri­phos­pho­rinane-2,4,6-trioxide (T3P)-promoted method (Silverberg *et al.*, 2021 ▸), a series of heteroaryl-substituted 2,3-di­hydro-4*H*-pyrido[3,2-*e*][1,3]thia­zin-4-ones are now being synthesized. In this communication, we report the synthesis and crystal structures of two compounds (**1** and **2**) in which there is a heteroaromatic ring on C2. Compound **1** has a 3-thio­phene and compound **2** has a 3-(1*H*)-indole. Thio­phene and indole derivatives are each known for their biological activity (da Cruz *et al.*, 2021 ▸; Konus *et al.*, 2022 ▸) and could have inter­esting effects on the activity of the 2-aryl-3-phenyl-2,3-di­hydro-4*H*-pyrido[3,2-*e*][1,3]thia­zin-4-ones. The new compounds each have a total of three heterocycles.
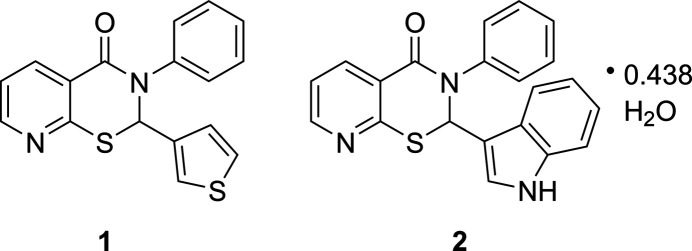


## 2. Structural commentary

**Figure 1:**
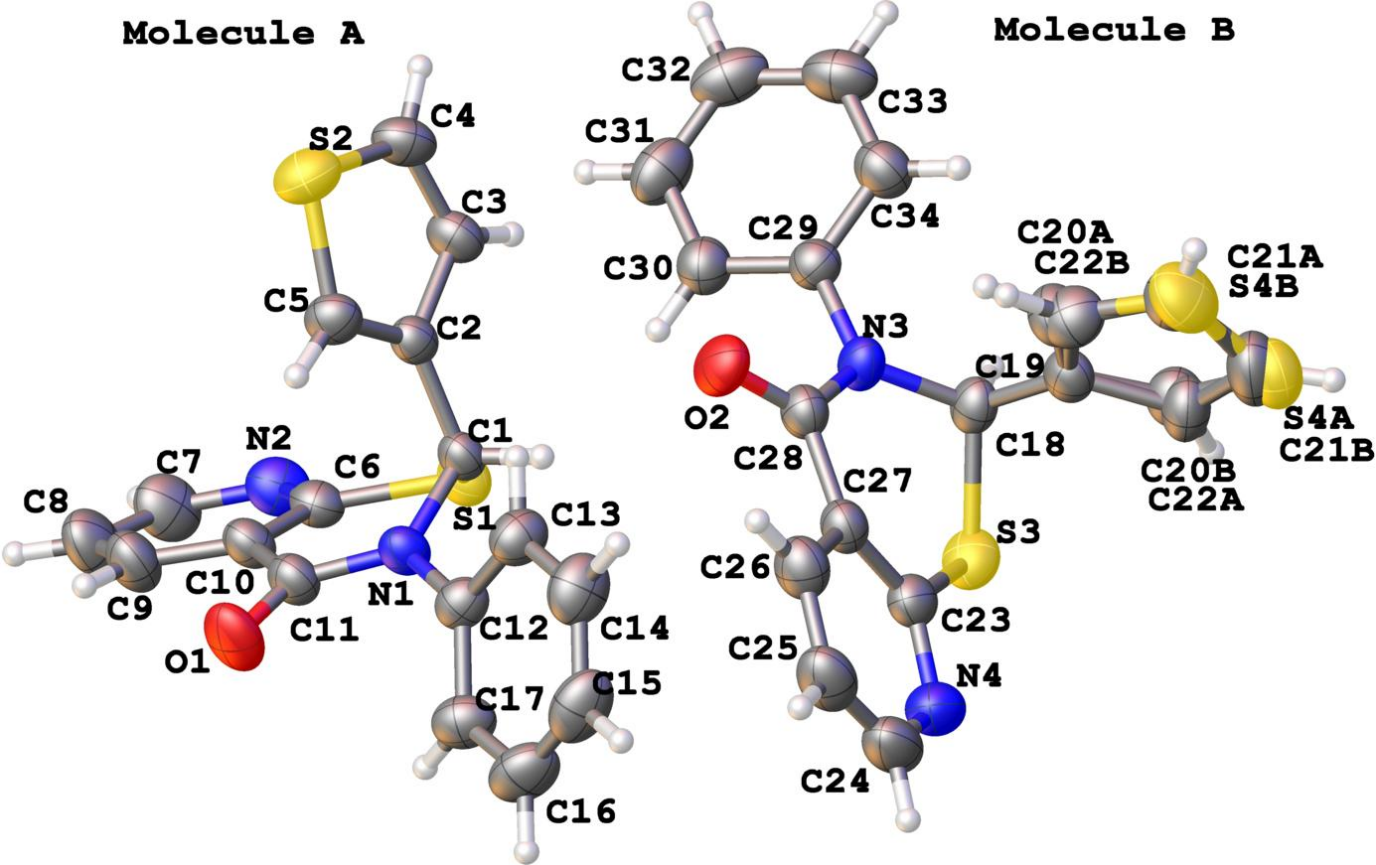
The mol­ecular structure of **1** with displacement ellipsoids drawn at the 50% probability level. Mol­ecules of both chirality are seen. The thio­phene ring of mol­ecule *B* exhibits a rotational disorder.

The title compounds crystallize in monoclinic lattices with two independent mol­ecules (*A* containing C1 in **1** and **2**, and *B* containing C18 in **1** and C22 in **2**; Figs. 1 ▸ and 2 ▸) in their respective asymmetric units. In **1**, mol­ecules of both chiralities are seen, while in **2** both mol­ecules of similar chirality occupy the asymmetric unit. In each structure the independent mol­ecules (with appropriate inversion applied in **1**) have almost identical configuration, as was confirmed by the alignment RMSD values falling within 0.013 Å when superposing chirally similar mol­ecules and matching the three non-H atoms surrounding the 2-carbon. In **2**, four disordered water mol­ecule sites were identified in difference-Fourier maps and refined well with manually adjusted quarter occupancy each. However, one of those oxygen atoms sits on a special position (multiplicity 2) resulting in a total contribution of 0.875 water mol­ecules per asymmetric unit (or about 0.438 water mol­ecules per parent mol­ecule). The core thia­zine ring in both structures exhibits an envelope conformation with the 2-carbon forming the flap [puckering amplitude *Q* ranging between 0.5545 (15) and 0.631 (2) Å, and the θ and φ values, after accounting for the absolute configuration transformations, are between 61.47 (17) and 66.50 (18)°, and 35.6 (2) and 47.14 (2)°, respectively].

## 3. Supra­molecular features

**Figure 3:**
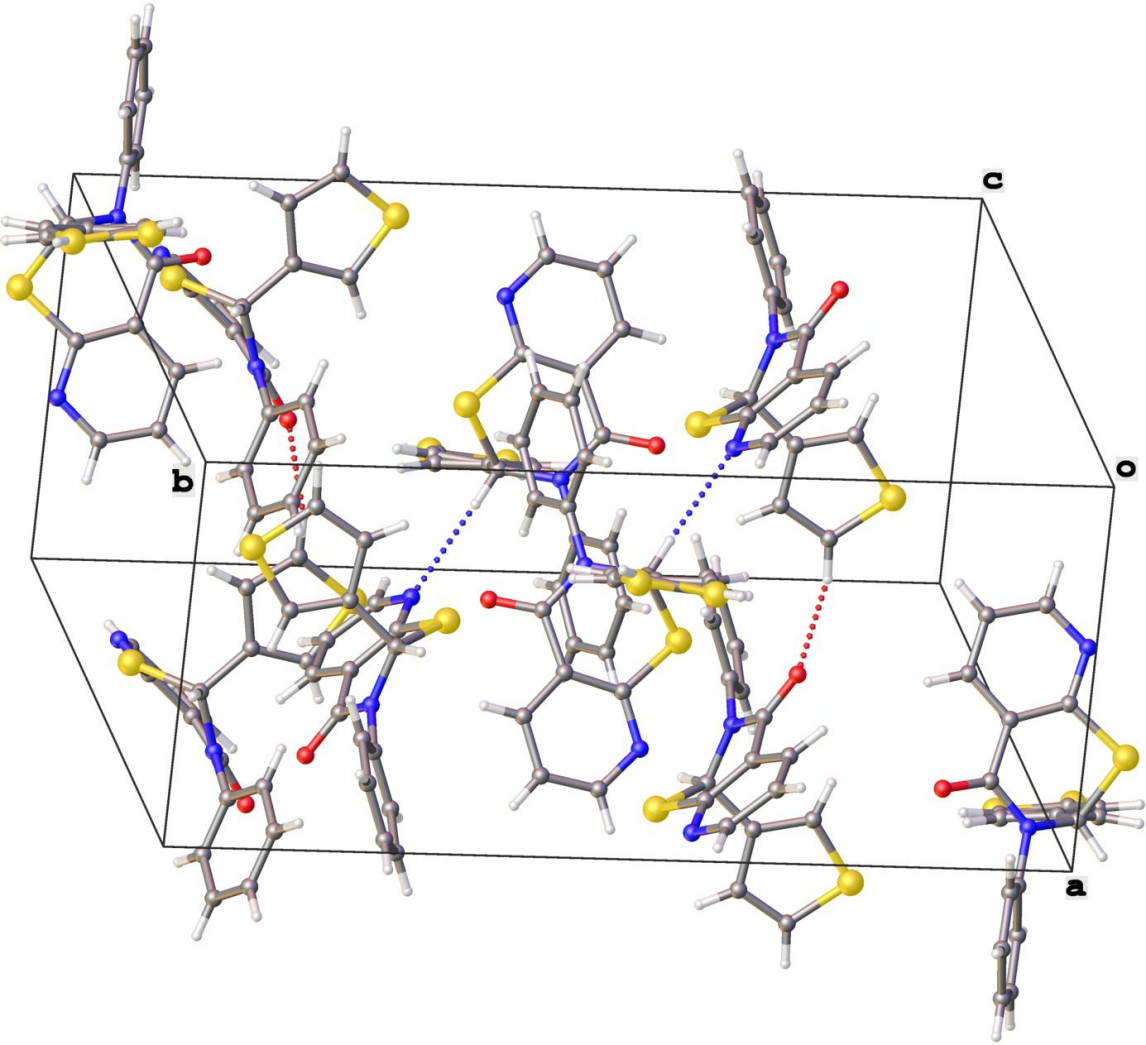
Packing diagram for **1** showing C—H⋯O and C—H⋯N type hydrogen bonds between mol­ecules.

In **1**, the inter­molecular inter­actions are defined solely by hydrogen bonds (Table 1 ▸, Fig. 3 ▸) of two types – the C—H⋯O type where a carbon atom of the thio­phene ring in mol­ecule *A* donates a proton to the only oxygen of its translational neighbour [C4—H4⋯O1 = 3.168 (2) Å, 164.5°] and the C—H⋯N type where the 2-carbon of the thia­zine in mol­ecule *B* donates a proton to the lone pair on the nitro­gen of fused pyridine ring of its independent neighbor *i.e.* mol­ecule *A* [C18—H18⋯N2 = 3.494 (2) Å, 167.1°]. The C—H⋯N type inter­actions are considered weak, but Webber *et al.* (2020 ▸) have studied their strengths. No π–π stacking inter­actions between rings of the neighboring mol­ecules were observed in this structure.

**Figure 4:**
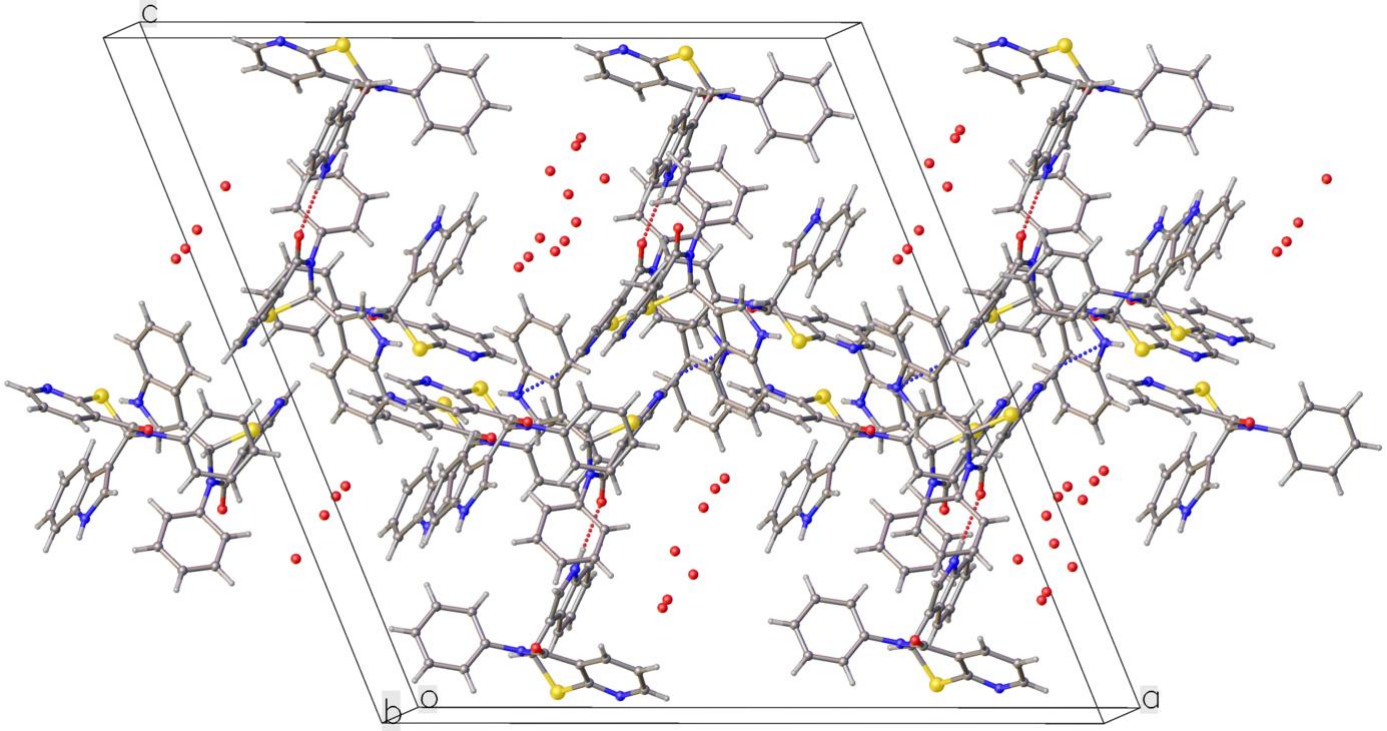
Packing diagram for **2** viewing down the *b*-axis, showing N—H⋯O and C—H⋯N hydrogen bonds between mol­ecules. The π–π stacking inter­actions that hold adjacent parallel chains akin to the teeth of a zipper and partially occupied water mol­ecules forming continuous channels down the *b-*axis are also seen.

In **2**, there are two types of hydrogen-bond inter­actions as well (Table 2 ▸, Fig. 4 ▸). One is an N—H⋯O type hydrogen bond [N3—H3⋯O2 = 2.828 (3) Å, 160.7°] where the nitro­gen of the indole ring of mol­ecule *A* donates a proton to the oxygen of enanti­omeric mol­ecule *B*. The other is a reciprocal pair of C—H⋯N(π) hydrogen bonds where a carbon from the fused pyridine ring donates a proton to the π electron cloud over the nitro­gen atom in the indole ring, connecting two enanti­omers of mol­ecule *B* in a give-and-take fashion [C26—H26⋯N6 = 3.463 (3) Å, 155.1°]. In the extended structure, the hydrogen bonds of both types result in the assembly of continuous mol­ecular chains in the [101] direction. Unlike the crystal of **1**, this one is further stabilized by π–π stacking inter­actions between pyridine rings of symmetry-related mol­ecules [the centroid–centroid distance and slippage are 3.5677 (16) and 1.017 Å, respectively]. These ring overlaps resemble the teeth of a zipper, binding the adjacent parallel mol­ecular chains. Continuous water channels along the *b*-axis direction punctuate the ‘teeth’, in an alternating fashion.

## 4. Database survey

We have previously reported crystal structures of 2,3-di­henyl-2,3-di­hydro-4*H*-pyrido[3,2-*e*][1,3]thia­zin-4-one (Yennawar *et al.*, 2014 ▸), its sulfoxide (Yennawar *et al.*, 2017 ▸), and its sulfone (Yennawar *et al.*, 2023 ▸). We have also reported structures of two 2-aryl-3-phenyl-2,3-di­hydro-4*H*-pyrido[3,2-*e*][1,3]thia­zin-4-ones, 2-(4-fluoro­phen­yl)-3-phenyl-2,3-di­hydro-4*H*-pyrido[3,2-*e*][1,3]thia­zin-4-one and 2-(4-nitro­phen­yl)-3-phenyl-2,3-di­hydro-4*H*-pyrido[3,2-*e*][1,3]thia­zin-4-one (Yennawar *et al.*, 2019 ▸).

## 5. Synthesis and crystallization

**General:** TLC plates (silica gel GF, 250-micron, 10 x 20 cm, cat. No. P21521) were purchased from Miles Scientific. TLCs were visualized under short wave UV, and then with I_2_, and then by spraying with ceric ammonium nitrate/sulfuric acid and heating. Infrared spectra were run on a Thermo-Fisher NICOLET iS50 FT-IR using a diamond-ATR attachment for direct powder analysis (Penn State Schuylkill). ^1^H and ^13^C NMR experiments (Penn State’s shared NMR facility, University Park) were carried out on a Bruker Avance-III-HD 500.20-MHz (^1^H frequency) instrument using a 5 mm Prodigy (liquid nitro­gen cooled) BBOBB-^1^H/^19^F/D Z-GRD cryo­probe. Samples were dissolved in pyridine-d5 and analyzed at RT. Typical conditions for ^1^H acquisition were 1 s relaxation delay, acquisition time of 3.28 s, and spectral width of 10 kHz, 32 scans. Spectra were zero-filled to 128k points, and multiplied by exponential multiplication (EM with LB = 0.3 Hz) prior to FT. For ^13^C experiments, data were acquired with power-gated ^1^H decoupling using a 2 s relaxation delay, with an acquisition time of 1.1 s, spectral width of 29.8 kHz, and 256 scans. Spectra were zero-filled once, and multiplied by EM with LB = 2 Hz prior to FT. MS samples were analyzed for purity and accurate mass by LCMS on a SCIEX Exion LC with a SCIEX 5600+ TripleTOF MS. Separation was achieved on an Agilent Infinity LabPoroshell column 120 EC-C18, 2.1 X 50mm, 2.7-micron particle (p/n 699775-902), column maintained at 313 K. Elution using a reversed phase gradient of 100% (water with 0.1% formic acid)ramped to 100% (aceto­nitrile with 0.1% formic acid) over 10 min at a flowrate of 0.4m L min^−1^. The MS was scanned over 50-1200 Da and calibrated with the SCIEX APCI positive calibrant solution (Part 4460131) prior to sample analysis. Samples were analyzed in ESI positive mode with a DP = 100 V, CE = 10, GAS1 = GAS2 = 60 psi, curtain = 30 psi, ISV = 5500 V, and source temperature of 773 K (Villanova University). Melting points were performed on a Vernier Melt Station (Penn State Schuylkill). Suitable crystals were selected and sequentially mounted using a nylon loop and a dab of paratone oil on a Rigaku Oxford diffraction, Synergy Custom system, HyPix-Arc 150 diffractometer at Penn State, University Park. The crystals were frozen to 173 (2) K during data collection. Using *OLEX2*, the structures were solved with the *SHELXT* (Sheldrick, 2015*a* ▸) structure solution program using Intrinsic Phasing and refined with the *SHELXL* (Sheldrick, 2015*b* ▸) refinement package using least-squares minimization.

**General Synthetic Procedure:** A two-necked 25 mL round-bottom flask was oven-dried, cooled under N_2_, and charged with a stirring bar. Aniline (0.559 g, 6 mmol) and a heteroaromatic aldehyde (3-thio­phencarboxaldehyde for **1** or 1*H*-indole-3-carbaldehyde for **2**; 6 mmol) was added. 2-Methyl­tetra­hydro­furan (2.3 mL) was added and the solution was stirred for five minutes. Thio­nicotinic acid (0.931 g, 6 mmol) was added. Pyridine (2.9 mL, 36 mmol) was added. Finally, 2,4,6-tripropyl-1,3,5,2,4,6-trioxatri­phospho­rinane-2,4,6-tri­oxide (T3P) in 2-methyl­tetra­hydro­furan (50 weight percent; 11 mL, 18 mmol) was added. The reaction was stirred at room temperature for 1–2 weeks and followed by TLC, then poured into a separatory funnel with di­chloro­methane (20 mL). The mixture was washed with water (10 mL). The aqueous solution was then extracted twice with di­chloro­methane (10 mL each). The organics were combined and washed with saturated sodium bicarbonate (10 mL) and then saturated sodium chloride (10 mL) solutions. The organic phase was dried over sodium sulfate and concentrated under vacuum to give a crude mixture. Further purification was carried out as indicated below for each compound.

**3-Phenyl-2-(thio­phen-3-yl)-2,3-di­hydro-4*****H*****-pyrido[3,2-*****e*****][1,3]thia­zin-4-one, 1**: After chromatography on 30 g silica gel with a gradient from 30% ethyl acetate / 70% hexa­nes to 70% ethyl acetate / 30% hexa­nes, recrystallization from 2-propanol, and then from ethyl acetate and hexa­nes gave an off-white powder (0.3456 g, 19% yield), m.p. 426.0-426.6 K. Crystals for crystallography were grown by slow evaporation from 2-propanol. ^1^H NMR (d5-pyridine) δ 8.56 (*d*, *J* = 7.9 Hz, 1H), 8.47 (*d*, *J* = 4.8 Hz, 1H), 7.61 (*d*, *J* = 8.2 Hz, 2H), 7.56 (*s* or *d* overlapping a solvent peak, 1H), 7.37 (*t*, *J* = 7.9 Hz, 2H), 7.34–7.29 (*m*, 1H), 7.28–7.21 (*m*, 2H), 7.06 (*dd*, *J* = 7.9, 4.7 Hz, 1H), 6.78 (*s*, 1H, S—CH—N). ^13^C NMR (d5-pyridine) δ 162.88 (C=O), 157.49, 152.88, 142.47, 141.50, 137.80, 129.28, 127.32, 127.20, 126.85, 126.33, 126.25, 124.01, 121.26, 61.85 (S—C—N). HRMS (*m*/*z*): [*M* + H^+^] of 325.0460 is consistent with calculated [*M* + H]^+^ of 325.0463. IR (neat, cm^−1^): 1641 (C=O).

**2-(1*****H*****-Indol-3-yl)-3-phenyl-2,3-di­hydro-4*****H*****-pyrido[3,2-*****e*****][1,3]thia­zin-4-one 0.438 hydrate, 2**: After chromatography on 30 g silica gel with a gradient from 30% ethyl acetate / 70% hexa­nes to 70% ethyl acetate / 30% hexa­nes, recrystallization from ethyl acetate and hexa­nes gave an off-white powder (0.855 g). ^1^H NMR showed this was an ethyl acetate solvate (mole ratio of 69.8% **2**: 30.2% ethyl acetate). Accounting for that, the yield of **2** was 0.772 g (36%), m.p. 416–418 K. Crystals for crystallography were grown by slow evaporation from ethanol. ^1^H NMR (d5-pyridine) δ 12.30 (*s*, 1H, NH), 8.60 (*dd*, *J* = 7.8, 2.0 Hz, 1H), 8.43 (*dd*, *J* = 4.7, 1.9 Hz, 1H), 8.08 (*d*, *J* = 7.0 Hz, 1H), 7.73 (*d*, *J* = 3.3 Hz, 1H), 7.68 (*d*, *J* = 8.2 Hz, 2H), 7.39 (*dd*, *J* = 6.2, 2.1 Hz, 1H), 7.32 (*t*, *J* = 7.9 Hz, 2H), 7.28–7.20 (*m*, 3H), 7.16 (*s*, 1H, S—CH—N), 7.02 (*dd*, *J* = 7.9, 4.7 Hz, 1H). ^13^C NMR (d5-pyridine) δ 163.30 (C=O), 158.41, 152.76, 142.64, 137.91, 137.83, 129.09, 126.95, 126.48, 126.26, 125.38, 125.22, 122.49, 120.96, 120.06, 119.86, 114.12, 112.21, 60.66 (S—C—N). [*M* + H^+^] of 358.1002 is consistent with calculated [*M* + H]^+^ of 358.1008. IR (neat, cm^−1^): 3245 (N—H), 1641 (C=O).

## 6. Refinement

Crystal data, data collection and structure refinement details are summarized in Table 3 ▸.

For refining structure **1** that has the rotational-flip of the thio­phene ring, rigid-group disorder, geometric and atomic-displacement restraints (RIGU, DFIX, SADI, SIMU, DELU and ISOR) were used to achieve the convergence.

The refinement of structure **2** involved four partially occupied water mol­ecules identified from the difference-Fourier map and their occupancies manually adjusted to 0.25 each. Placing the hydrogen atoms on the water mol­ecules resulted in high shift/esd values and so were excluded. ISOR restraint for all the four water oxygens and for C10, C11 atoms of phenyl ring in mol­ecule *A* and C38 and C39 atoms in the indole ring of mol­ecule *B*, as well as SIMU and DELU for all atoms in the structure, helped converge the refinement. In both above structures, hydrogen atoms were placed at calculated positions and refined using a riding model.

## Supplementary Material

Crystal structure: contains datablock(s) 1, 2. DOI: 10.1107/S2056989024005103/tx2086sup1.cif

Structure factors: contains datablock(s) 1. DOI: 10.1107/S2056989024005103/tx20861sup2.hkl

Supporting information file. DOI: 10.1107/S2056989024005103/tx20861sup4.mol

Structure factors: contains datablock(s) 2. DOI: 10.1107/S2056989024005103/tx20862sup3.hkl

Supporting information file. DOI: 10.1107/S2056989024005103/tx20862sup5.mol

Supporting information file. DOI: 10.1107/S2056989024005103/tx20861sup6.cml

Supporting information file. DOI: 10.1107/S2056989024005103/tx20862sup7.cml

CCDC references: 2359143, 2359142

Additional supporting information:  crystallographic information; 3D view; checkCIF report

## Figures and Tables

**Figure 2:**
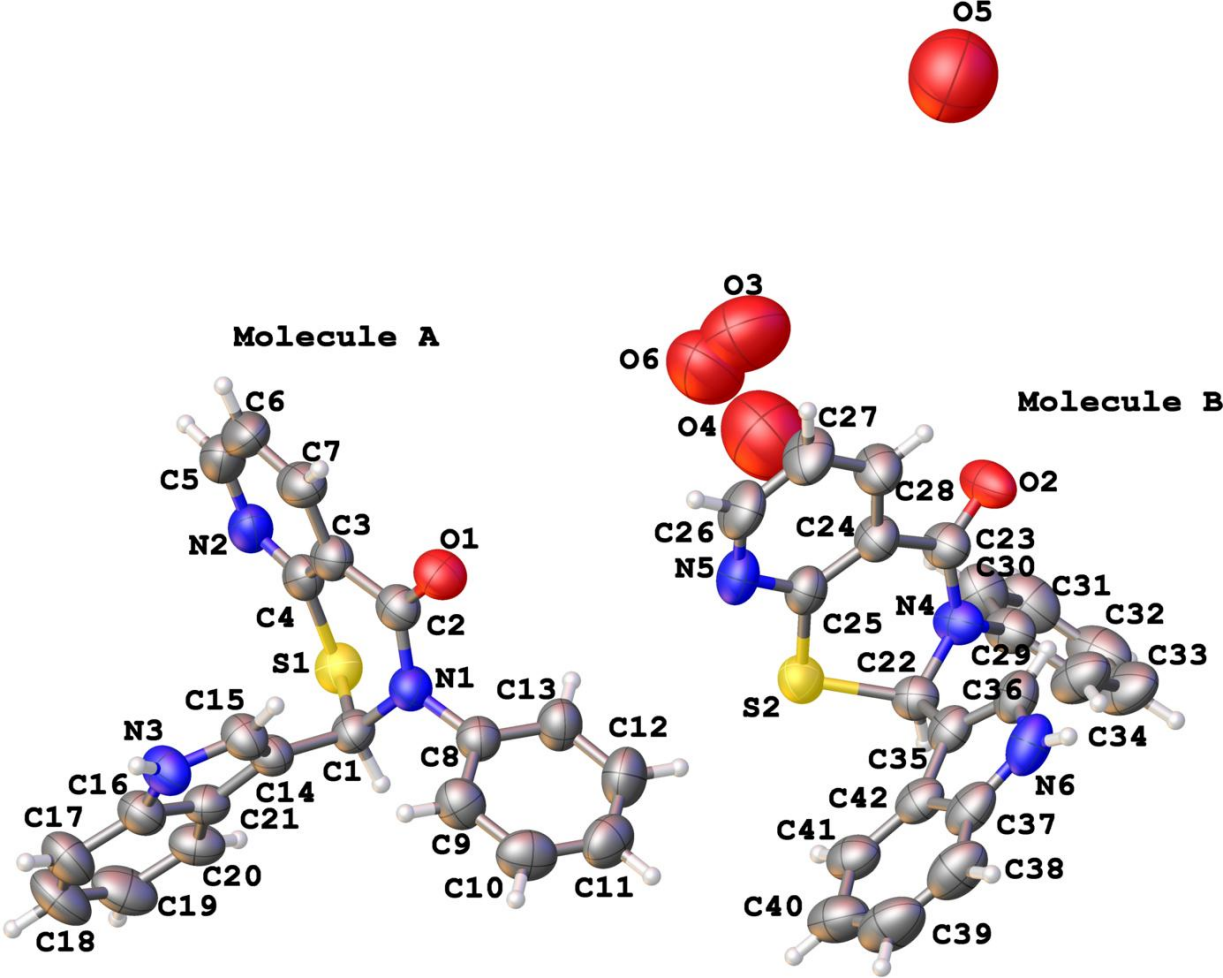
The mol­ecular structure of **2** with displacement ellipsoids drawn at the 50% probability level. Both mol­ecules have the same chirality. The water O atoms (O3 to O6) at quarter occupancy each were refined without protons.

**Table 1:** Hydrogen-bond geometry (Å, °) for **1**[Chem scheme1]

*D*—H⋯*A*	*D*—H	H⋯*A*	*D*⋯*A*	*D*—H⋯*A*
C4—H4⋯O1^i^	0.93	2.26	3.168 (2)	165
C18—H18⋯N2^ii^	0.98	2.53	3.494 (2)	167

Symmetry codes: (i) 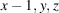; (ii) 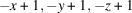.

**Table 2:** Hydrogen-bond geometry (Å, °) for **2**[Chem scheme1]

*D*—H⋯*A*	*D*—H	H⋯*A*	*D*⋯*A*	*D*—H⋯*A*
N3—H3⋯O2^i^	0.86	2.00	2.828 (3)	161
C26—H26⋯N6^ii^	0.93	2.60	3.463 (3)	155

Symmetry codes: (i) 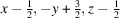; (ii) 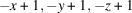.

**Table 3:** Experimental details

	**1**	**2**
Crystal data		
Chemical formula	C_17_H_12_N_2_OS_2_	C_21_H_15_N_3_OS·0.438H_2_O
*M*_r_	324.41	364.85
Crystal system, space group	Monoclinic, *P*2_1_/*n*	Monoclinic, *C*2/*c*
Temperature (K)	173	173
*a*, *b*, *c* (Å)	9.22549 (10), 20.8037 (2), 15.87594 (19)	28.2424 (4), 11.0307 (1), 28.6936 (4)
β (°)	92.7309 (11)	111.952 (2)
*V* (Å^3^)	3043.52 (6)	8290.9 (2)
*Z*	8	16
Radiation type	Cu *K*α	Cu *K*α
μ (mm^−1^)	3.19	1.51
Crystal size (mm)	0.15 × 0.13 × 0.08	0.2 × 0.2 × 0.17

Data collection		
Diffractometer	ROD, Synergy Custom system, HyPix-Arc 150	ROD, Synergy Custom system, HyPix-Arc 150
Absorption correction	Multi-scan (*CrysAlis PRO*; Rigaku OD, 2023 ▸)	Multi-scan (*CrysAlis PRO*; Rigaku OD, 2023 ▸)
*T*_min_, *T*_max_	0.628, 1.000	0.652, 1.000
No. of measured, independent and observed [*I* > 2σ(*I*)] reflections	20274, 6042, 4922	26357, 8118, 6592
*R*_int_	0.032	0.027
(sin θ/λ)_max_ (Å^−1^)	0.630	0.630

Refinement		
*R*[*F*^2^ > 2σ(*F*^2^)], *wR*(*F*^2^), *S*	0.035, 0.096, 1.07	0.068, 0.218, 1.09
No. of reflections	6042	8118
No. of parameters	435	492
No. of restraints	139	560
H-atom treatment	H-atom parameters constrained	H-atom parameters constrained
Δρ_max_, Δρ_min_ (e Å^−3^)	0.20, −0.32	0.93, −0.25

Computer programs: *CrysAlis PRO* (Rigaku OD, 2023 ▸), *SHELXT* (Sheldrick, 2015*a* ▸), *SHELXL2018/3* (Sheldrick, 2015*b* ▸) and *OLEX2* (Dolomanov *et al.*, 2009 ▸).

## supplementary crystallographic information

### 3-Phenyl-2-(thiophen-3-yl)-2,3-dihydro-4H-pyrido[3,2-e][1,3]thiazin-4-one (1) Crystal data

**Table:**

C_17_H_12_N_2_OS_2_	*F*(000) = 1344
*M**_r_* = 324.41	*D*_x_ = 1.416 Mg m^−^^3^
Monoclinic, *P*2_1_/*n*	Cu *K*α radiation, λ = 1.54184 Å
*a* = 9.22549 (10) Å	Cell parameters from 9971 reflections
*b* = 20.8037 (2) Å	θ = 3.5–76.2°
*c* = 15.87594 (19) Å	µ = 3.19 mm^−^^1^
β = 92.7309 (11)°	*T* = 173 K
*V* = 3043.52 (6) Å^3^	Block, clear colourless
*Z* = 8	0.15 × 0.13 × 0.08 mm

### 3-Phenyl-2-(thiophen-3-yl)-2,3-dihydro-4H-pyrido[3,2-e][1,3]thiazin-4-one (1) Data collection

**Table:**

ROD, Synergy Custom system, HyPix-Arc 150 diffractometer	6042 independent reflections
Radiation source: Rotating-anode X-ray tube, Rigaku (Cu) X-ray Source	4922 reflections with *I* > 2σ(*I*)
Mirror monochromator	*R*_int_ = 0.032
Detector resolution: 10.0000 pixels mm^-1^	θ_max_ = 76.4°, θ_min_ = 3.5°
ω scans	*h* = −11→11
Absorption correction: multi-scan (CrysAlisPro; Rigaku OD, 2023)	*k* = −24→25
*T*_min_ = 0.628, *T*_max_ = 1.000	*l* = −19→16
20274 measured reflections	

### 3-Phenyl-2-(thiophen-3-yl)-2,3-dihydro-4H-pyrido[3,2-e][1,3]thiazin-4-one (1) Refinement

**Table:**

Refinement on *F*^2^	Hydrogen site location: inferred from neighbouring sites
Least-squares matrix: full	H-atom parameters constrained
*R*[*F*^2^ > 2σ(*F*^2^)] = 0.035	*w* = 1/[σ^2^(*F*_o_^2^) + (0.0423*P*)^2^ + 0.5556*P*] where *P* = (*F*_o_^2^ + 2*F*_c_^2^)/3
*wR*(*F*^2^) = 0.096	(Δ/σ)_max_ = 0.001
*S* = 1.07	Δρ_max_ = 0.20 e Å^−^^3^
6042 reflections	Δρ_min_ = −0.32 e Å^−^^3^
435 parameters	Extinction correction: *SHELXL2018/3* (Sheldrick, 2015b), Fc^*^=kFc[1+0.001xFc^2^λ^3^/sin(2θ)]^-1/4^
139 restraints	Extinction coefficient: 0.00078 (8)
Primary atom site location: dual	

### 3-Phenyl-2-(thiophen-3-yl)-2,3-dihydro-4H-pyrido[3,2-e][1,3]thiazin-4-one (1) Special details

**Table:**

Geometry. All esds (except the esd in the dihedral angle between two l.s. planes) are estimated using the full covariance matrix. The cell esds are taken into account individually in the estimation of esds in distances, angles and torsion angles; correlations between esds in cell parameters are only used when they are defined by crystal symmetry. An approximate (isotropic) treatment of cell esds is used for estimating esds involving l.s. planes.

### 3-Phenyl-2-(thiophen-3-yl)-2,3-dihydro-4H-pyrido[3,2-e][1,3]thiazin-4-one (1) Fractional atomic coordinates and isotropic or equivalent isotropic displacement parameters (Å^2^)

**Table:**

	*x*	*y*	*z*	*U*_iso_*/*U*_eq_	Occ. (<1)
S1	0.77155 (5)	0.62406 (2)	0.51130 (3)	0.04325 (12)	
S2	0.56507 (6)	0.85356 (2)	0.47059 (3)	0.05474 (14)	
O1	1.12238 (14)	0.76514 (8)	0.50000 (9)	0.0611 (4)	
N1	0.93720 (14)	0.71164 (7)	0.43232 (9)	0.0383 (3)	
N2	0.83219 (17)	0.63711 (8)	0.67292 (10)	0.0514 (4)	
C1	0.78696 (17)	0.69009 (8)	0.43630 (11)	0.0365 (4)	
H1	0.758821	0.672688	0.380531	0.044*	
C2	0.68234 (17)	0.74370 (8)	0.45268 (10)	0.0350 (3)	
C3	0.53153 (18)	0.73296 (9)	0.46130 (12)	0.0445 (4)	
H3	0.488994	0.692420	0.459691	0.053*	
C4	0.4561 (2)	0.78833 (10)	0.47216 (13)	0.0503 (5)	
H4	0.356762	0.790048	0.479538	0.060*	
C5	0.71511 (19)	0.80735 (9)	0.45651 (12)	0.0440 (4)	
H5	0.808150	0.823803	0.452003	0.053*	
C6	0.86214 (18)	0.66143 (8)	0.59760 (11)	0.0395 (4)	
C7	0.9010 (2)	0.66294 (12)	0.74017 (13)	0.0619 (6)	
H7	0.883201	0.645858	0.792814	0.074*	
C8	0.9969 (2)	0.71339 (13)	0.73668 (13)	0.0650 (6)	
H8	1.039798	0.730783	0.785748	0.078*	
C9	1.0278 (2)	0.73741 (11)	0.65925 (13)	0.0553 (5)	
H9	1.092899	0.771252	0.655162	0.066*	
C10	0.96128 (17)	0.71101 (9)	0.58664 (11)	0.0410 (4)	
C11	1.01260 (18)	0.73236 (9)	0.50360 (11)	0.0420 (4)	
C12	0.99721 (18)	0.71782 (8)	0.35064 (11)	0.0399 (4)	
C13	0.9161 (2)	0.74399 (9)	0.28410 (12)	0.0490 (4)	
H13	0.823682	0.759897	0.292406	0.059*	
C14	0.9723 (3)	0.74662 (10)	0.20480 (13)	0.0602 (5)	
H14	0.917104	0.763988	0.159829	0.072*	
C15	1.1088 (3)	0.72372 (11)	0.19240 (14)	0.0633 (6)	
H15	1.146461	0.725768	0.139154	0.076*	
C16	1.1901 (3)	0.69772 (11)	0.25872 (15)	0.0625 (6)	
H16	1.282569	0.682033	0.249955	0.075*	
C17	1.1359 (2)	0.69463 (9)	0.33842 (13)	0.0501 (5)	
H17	1.191497	0.677257	0.383208	0.060*	
S3	0.62400 (6)	0.41208 (2)	0.26334 (3)	0.05086 (14)	
S4B	0.2995 (2)	0.50603 (6)	0.02355 (7)	0.0711 (3)	0.874 (3)
O2	0.56355 (15)	0.61714 (6)	0.30744 (9)	0.0556 (4)	
N3	0.46385 (15)	0.51735 (7)	0.30055 (9)	0.0412 (3)	
N4	0.85514 (18)	0.45429 (9)	0.19311 (11)	0.0585 (4)	
C18	0.45275 (19)	0.45587 (8)	0.25591 (11)	0.0414 (4)	
H18	0.381763	0.429829	0.284727	0.050*	
C19	0.39720 (18)	0.46234 (9)	0.16534 (11)	0.0428 (4)	
C20B	0.3787 (10)	0.4084 (3)	0.1097 (3)	0.0535 (12)	0.874 (3)
H20B	0.400998	0.366169	0.124316	0.064*	0.874 (3)
C21B	0.3241 (12)	0.4275 (5)	0.0325 (6)	0.075 (3)	0.874 (3)
H21B	0.302562	0.398858	−0.011382	0.090*	0.874 (3)
C22B	0.3586 (10)	0.5181 (2)	0.1271 (3)	0.0529 (10)	0.874 (3)
H22B	0.363219	0.558111	0.153342	0.063*	0.874 (3)
C23	0.73266 (19)	0.47405 (9)	0.22665 (11)	0.0431 (4)	
C24	0.9417 (2)	0.49976 (13)	0.16421 (14)	0.0644 (6)	
H24	1.027172	0.486821	0.140496	0.077*	
C25	0.9128 (2)	0.56440 (12)	0.16714 (13)	0.0616 (6)	
H25	0.976064	0.594185	0.145228	0.074*	
C26	0.7870 (2)	0.58404 (10)	0.20361 (12)	0.0521 (5)	
H26	0.765099	0.627564	0.207161	0.062*	
C27	0.69361 (18)	0.53857 (8)	0.23486 (11)	0.0407 (4)	
C28	0.56838 (18)	0.56141 (8)	0.28296 (11)	0.0417 (4)	
C29	0.35521 (18)	0.53267 (8)	0.35951 (11)	0.0398 (4)	
C30	0.3959 (2)	0.55445 (9)	0.43908 (12)	0.0480 (4)	
H30	0.493426	0.560768	0.454313	0.058*	
C31	0.2907 (3)	0.56686 (10)	0.49621 (13)	0.0587 (5)	
H31	0.318003	0.581916	0.549804	0.070*	
C32	0.1466 (3)	0.55717 (11)	0.47459 (15)	0.0631 (6)	
H32	0.076454	0.565609	0.513315	0.076*	
C33	0.1067 (2)	0.53507 (11)	0.39593 (16)	0.0629 (6)	
H33	0.009141	0.528030	0.381416	0.076*	
C34	0.2101 (2)	0.52305 (10)	0.33751 (13)	0.0522 (5)	
H34	0.182062	0.508564	0.283761	0.063*	
C20A	0.357 (7)	0.5169 (16)	0.112 (2)	0.057 (6)	0.126 (3)
H20A	0.370954	0.559347	0.128254	0.068*	0.126 (3)
C21A	0.298 (6)	0.5006 (12)	0.037 (2)	0.064 (5)	0.126 (3)
H21A	0.250599	0.528811	0.000010	0.077*	0.126 (3)
S4A	0.322 (2)	0.4206 (8)	0.0199 (10)	0.063 (3)	0.126 (3)
C22A	0.367 (7)	0.4094 (17)	0.123 (2)	0.051 (5)	0.126 (3)
H22A	0.369269	0.368919	0.148137	0.061*	0.126 (3)

### 3-Phenyl-2-(thiophen-3-yl)-2,3-dihydro-4H-pyrido[3,2-e][1,3]thiazin-4-one (1) Atomic displacement parameters (Å^2^)

**Table:**

	*U*^11^	*U*^22^	*U*^33^	*U*^12^	*U*^13^	*U*^23^
S1	0.0401 (2)	0.0375 (2)	0.0518 (3)	−0.00712 (17)	−0.00127 (19)	0.00235 (19)
S2	0.0566 (3)	0.0460 (3)	0.0630 (3)	0.0070 (2)	0.0158 (2)	0.0029 (2)
O1	0.0429 (7)	0.0816 (10)	0.0591 (8)	−0.0285 (7)	0.0060 (6)	−0.0032 (8)
N1	0.0306 (7)	0.0475 (8)	0.0372 (7)	−0.0045 (6)	0.0050 (6)	0.0003 (6)
N2	0.0507 (9)	0.0552 (9)	0.0485 (9)	0.0004 (7)	0.0036 (8)	0.0120 (8)
C1	0.0301 (8)	0.0422 (9)	0.0371 (9)	−0.0047 (7)	0.0006 (7)	−0.0024 (7)
C2	0.0324 (8)	0.0418 (9)	0.0307 (8)	−0.0023 (7)	0.0011 (6)	0.0005 (7)
C3	0.0338 (8)	0.0488 (10)	0.0507 (11)	−0.0053 (7)	0.0015 (8)	0.0032 (8)
C4	0.0344 (9)	0.0624 (12)	0.0544 (11)	0.0026 (8)	0.0069 (8)	0.0050 (10)
C5	0.0377 (9)	0.0452 (10)	0.0499 (11)	−0.0028 (7)	0.0100 (8)	0.0004 (8)
C6	0.0334 (8)	0.0414 (9)	0.0435 (10)	0.0018 (7)	0.0006 (7)	0.0030 (8)
C7	0.0629 (13)	0.0808 (15)	0.0416 (11)	−0.0014 (11)	−0.0010 (10)	0.0123 (11)
C8	0.0564 (12)	0.0964 (18)	0.0414 (11)	−0.0060 (12)	−0.0071 (10)	−0.0028 (11)
C9	0.0423 (10)	0.0729 (14)	0.0504 (11)	−0.0132 (9)	−0.0021 (9)	−0.0055 (10)
C10	0.0315 (8)	0.0502 (10)	0.0410 (9)	−0.0035 (7)	−0.0013 (7)	0.0013 (8)
C11	0.0329 (8)	0.0482 (10)	0.0447 (10)	−0.0062 (7)	0.0004 (7)	−0.0013 (8)
C12	0.0412 (9)	0.0379 (9)	0.0415 (9)	−0.0030 (7)	0.0103 (8)	0.0003 (7)
C13	0.0539 (11)	0.0501 (10)	0.0435 (10)	0.0049 (9)	0.0068 (9)	0.0002 (8)
C14	0.0810 (15)	0.0568 (12)	0.0433 (11)	−0.0016 (11)	0.0082 (10)	0.0041 (9)
C15	0.0816 (16)	0.0588 (13)	0.0522 (12)	−0.0119 (11)	0.0304 (12)	−0.0044 (10)
C16	0.0570 (12)	0.0608 (13)	0.0721 (15)	−0.0019 (10)	0.0295 (11)	−0.0034 (11)
C17	0.0435 (10)	0.0504 (11)	0.0575 (12)	0.0000 (8)	0.0129 (9)	0.0043 (9)
S3	0.0598 (3)	0.0378 (2)	0.0546 (3)	0.0023 (2)	−0.0010 (2)	0.0007 (2)
S4B	0.0671 (5)	0.0914 (6)	0.0535 (5)	−0.0006 (4)	−0.0101 (5)	0.0081 (4)
O2	0.0558 (8)	0.0390 (7)	0.0727 (9)	−0.0084 (6)	0.0106 (7)	−0.0143 (6)
N3	0.0405 (8)	0.0389 (7)	0.0448 (8)	−0.0080 (6)	0.0068 (6)	−0.0090 (6)
N4	0.0457 (9)	0.0692 (11)	0.0606 (11)	0.0073 (8)	0.0024 (8)	−0.0125 (9)
C18	0.0451 (9)	0.0367 (9)	0.0428 (10)	−0.0091 (7)	0.0057 (8)	−0.0060 (7)
C19	0.0369 (9)	0.0457 (9)	0.0461 (10)	−0.0082 (7)	0.0039 (8)	−0.0049 (8)
C20B	0.058 (3)	0.0543 (15)	0.0480 (19)	−0.0117 (14)	0.003 (2)	−0.0131 (14)
C21B	0.072 (4)	0.091 (4)	0.061 (3)	−0.014 (3)	−0.001 (2)	−0.025 (2)
C22B	0.0563 (18)	0.0554 (16)	0.046 (2)	−0.0025 (14)	−0.005 (2)	−0.0014 (14)
C23	0.0417 (9)	0.0484 (10)	0.0389 (9)	0.0007 (8)	−0.0021 (8)	−0.0041 (8)
C24	0.0413 (11)	0.0929 (18)	0.0591 (13)	−0.0019 (11)	0.0047 (10)	−0.0168 (13)
C25	0.0471 (11)	0.0833 (16)	0.0551 (12)	−0.0183 (11)	0.0089 (10)	−0.0064 (11)
C26	0.0514 (11)	0.0541 (11)	0.0508 (11)	−0.0117 (9)	0.0026 (9)	−0.0019 (9)
C27	0.0383 (9)	0.0440 (9)	0.0396 (9)	−0.0062 (7)	−0.0006 (7)	−0.0038 (8)
C28	0.0403 (9)	0.0407 (9)	0.0439 (10)	−0.0054 (7)	0.0003 (8)	−0.0058 (8)
C29	0.0389 (9)	0.0385 (9)	0.0421 (9)	−0.0010 (7)	0.0041 (7)	−0.0011 (7)
C30	0.0496 (10)	0.0489 (10)	0.0454 (10)	−0.0013 (8)	0.0011 (9)	−0.0025 (9)
C31	0.0770 (15)	0.0571 (12)	0.0430 (11)	0.0057 (11)	0.0123 (10)	−0.0012 (9)
C32	0.0664 (14)	0.0541 (12)	0.0713 (15)	0.0109 (10)	0.0291 (12)	0.0103 (11)
C33	0.0416 (11)	0.0655 (14)	0.0824 (16)	0.0014 (9)	0.0111 (11)	0.0096 (12)
C34	0.0440 (10)	0.0577 (12)	0.0547 (12)	−0.0049 (9)	−0.0005 (9)	−0.0014 (10)
C20A	0.061 (11)	0.047 (7)	0.063 (8)	−0.001 (7)	0.007 (7)	0.004 (5)
C21A	0.064 (5)	0.064 (5)	0.064 (5)	−0.0001 (10)	0.0031 (10)	0.0004 (10)
S4A	0.062 (5)	0.067 (4)	0.062 (5)	−0.015 (3)	−0.002 (3)	−0.008 (4)
C22A	0.051 (10)	0.052 (6)	0.050 (7)	−0.006 (6)	0.007 (8)	−0.002 (5)

### 3-Phenyl-2-(thiophen-3-yl)-2,3-dihydro-4H-pyrido[3,2-e][1,3]thiazin-4-one (1) Geometric parameters (Å, º)

**Table:**

S1—C1	1.8280 (17)	O2—C28	1.224 (2)
S1—C6	1.7524 (18)	N3—C18	1.463 (2)
S2—C4	1.690 (2)	N3—C28	1.369 (2)
S2—C5	1.7086 (18)	N3—C29	1.439 (2)
O1—C11	1.225 (2)	N4—C23	1.337 (2)
N1—C1	1.461 (2)	N4—C24	1.333 (3)
N1—C11	1.369 (2)	C18—H18	0.9800
N1—C12	1.440 (2)	C18—C19	1.509 (2)
N2—C6	1.339 (2)	C19—C20B	1.433 (5)
N2—C7	1.329 (3)	C19—C22B	1.350 (5)
C1—H1	0.9800	C19—C20A	1.46 (3)
C1—C2	1.506 (2)	C19—C22A	1.31 (3)
C2—C3	1.422 (2)	C20B—H20B	0.9300
C2—C5	1.359 (2)	C20B—C21B	1.362 (10)
C3—H3	0.9300	C21B—H21B	0.9300
C3—C4	1.361 (3)	C22B—H22B	0.9300
C4—H4	0.9300	C23—C27	1.398 (2)
C5—H5	0.9300	C24—H24	0.9300
C6—C10	1.395 (2)	C24—C25	1.372 (3)
C7—H7	0.9300	C25—H25	0.9300
C7—C8	1.376 (3)	C25—C26	1.383 (3)
C8—H8	0.9300	C26—H26	0.9300
C8—C9	1.369 (3)	C26—C27	1.387 (2)
C9—H9	0.9300	C27—C28	1.492 (2)
C9—C10	1.393 (3)	C29—C30	1.377 (2)
C10—C11	1.490 (2)	C29—C34	1.382 (2)
C12—C13	1.377 (3)	C30—H30	0.9300
C12—C17	1.389 (2)	C30—C31	1.384 (3)
C13—H13	0.9300	C31—H31	0.9300
C13—C14	1.385 (3)	C31—C32	1.373 (3)
C14—H14	0.9300	C32—H32	0.9300
C14—C15	1.370 (3)	C32—C33	1.365 (3)
C15—H15	0.9300	C33—H33	0.9300
C15—C16	1.374 (3)	C33—C34	1.385 (3)
C16—H16	0.9300	C34—H34	0.9300
C16—C17	1.384 (3)	C20A—H20A	0.9300
C17—H17	0.9300	C20A—C21A	1.32 (3)
S3—C18	1.8226 (19)	C21A—H21A	0.9300
S3—C23	1.7498 (19)	C21A—S4A	1.703 (17)
S4B—C21B	1.655 (9)	S4A—C22A	1.69 (3)
S4B—C22B	1.726 (4)	C22A—H22A	0.9300
			
C6—S1—C1	97.29 (8)	N3—C18—S3	111.41 (12)
C4—S2—C5	92.08 (9)	N3—C18—H18	106.9
C11—N1—C1	120.42 (14)	N3—C18—C19	113.34 (15)
C11—N1—C12	120.95 (14)	C19—C18—S3	111.11 (12)
C12—N1—C1	118.21 (13)	C19—C18—H18	106.9
C7—N2—C6	116.96 (18)	C20B—C19—C18	122.9 (3)
S1—C1—H1	106.6	C22B—C19—C18	125.1 (2)
N1—C1—S1	111.23 (11)	C22B—C19—C20B	112.0 (3)
N1—C1—H1	106.6	C20A—C19—C18	133.9 (13)
N1—C1—C2	113.43 (13)	C22A—C19—C18	117.8 (14)
C2—C1—S1	111.94 (11)	C22A—C19—C20A	108.2 (19)
C2—C1—H1	106.6	C19—C20B—H20B	124.6
C3—C2—C1	122.58 (15)	C21B—C20B—C19	110.8 (6)
C5—C2—C1	125.90 (15)	C21B—C20B—H20B	124.6
C5—C2—C3	111.43 (16)	S4B—C21B—H21B	123.0
C2—C3—H3	123.6	C20B—C21B—S4B	114.0 (6)
C4—C3—C2	112.82 (16)	C20B—C21B—H21B	123.0
C4—C3—H3	123.6	S4B—C22B—H22B	124.2
S2—C4—H4	124.2	C19—C22B—S4B	111.6 (3)
C3—C4—S2	111.69 (14)	C19—C22B—H22B	124.2
C3—C4—H4	124.2	N4—C23—S3	114.50 (15)
S2—C5—H5	124.0	N4—C23—C27	124.00 (18)
C2—C5—S2	111.97 (13)	C27—C23—S3	121.48 (14)
C2—C5—H5	124.0	N4—C24—H24	117.9
N2—C6—S1	114.80 (13)	N4—C24—C25	124.3 (2)
N2—C6—C10	123.72 (16)	C25—C24—H24	117.9
C10—C6—S1	121.44 (13)	C24—C25—H25	120.9
N2—C7—H7	118.0	C24—C25—C26	118.1 (2)
N2—C7—C8	124.0 (2)	C26—C25—H25	120.9
C8—C7—H7	118.0	C25—C26—H26	120.1
C7—C8—H8	120.8	C25—C26—C27	119.7 (2)
C9—C8—C7	118.4 (2)	C27—C26—H26	120.1
C9—C8—H8	120.8	C23—C27—C28	124.25 (16)
C8—C9—H9	120.1	C26—C27—C23	117.02 (17)
C8—C9—C10	119.81 (19)	C26—C27—C28	118.36 (17)
C10—C9—H9	120.1	O2—C28—N3	122.19 (17)
C6—C10—C11	124.67 (16)	O2—C28—C27	120.31 (16)
C9—C10—C6	116.99 (17)	N3—C28—C27	117.47 (15)
C9—C10—C11	117.97 (16)	C30—C29—N3	120.11 (15)
O1—C11—N1	121.69 (17)	C30—C29—C34	119.85 (17)
O1—C11—C10	120.39 (16)	C34—C29—N3	119.98 (16)
N1—C11—C10	117.86 (14)	C29—C30—H30	120.2
C13—C12—N1	120.53 (15)	C29—C30—C31	119.59 (18)
C13—C12—C17	120.02 (17)	C31—C30—H30	120.2
C17—C12—N1	119.39 (16)	C30—C31—H31	119.7
C12—C13—H13	120.0	C32—C31—C30	120.7 (2)
C12—C13—C14	119.98 (19)	C32—C31—H31	119.7
C14—C13—H13	120.0	C31—C32—H32	120.2
C13—C14—H14	119.9	C33—C32—C31	119.6 (2)
C15—C14—C13	120.2 (2)	C33—C32—H32	120.2
C15—C14—H14	119.9	C32—C33—H33	119.7
C14—C15—H15	120.0	C32—C33—C34	120.6 (2)
C14—C15—C16	119.9 (2)	C34—C33—H33	119.7
C16—C15—H15	120.0	C29—C34—C33	119.65 (19)
C15—C16—H16	119.6	C29—C34—H34	120.2
C15—C16—C17	120.7 (2)	C33—C34—H34	120.2
C17—C16—H16	119.6	C19—C20A—H20A	123.0
C12—C17—H17	120.4	C21A—C20A—C19	114 (2)
C16—C17—C12	119.1 (2)	C21A—C20A—H20A	123.0
C16—C17—H17	120.4	C20A—C21A—H21A	124.8
C23—S3—C18	96.82 (8)	C20A—C21A—S4A	110 (2)
C21B—S4B—C22B	91.5 (3)	S4A—C21A—H21A	124.8
C28—N3—C18	121.34 (14)	C22A—S4A—C21A	90.2 (16)
C28—N3—C29	120.23 (14)	C19—C22A—S4A	115 (2)
C29—N3—C18	118.28 (13)	C19—C22A—H22A	122.7
C24—N4—C23	116.76 (19)	S4A—C22A—H22A	122.7
S3—C18—H18	106.9		
			
S1—C1—C2—C3	−51.29 (19)	N3—C18—C19—C20B	179.5 (5)
S1—C1—C2—C5	132.55 (16)	N3—C18—C19—C22B	−0.8 (5)
S1—C6—C10—C9	−179.84 (14)	N3—C18—C19—C20A	2 (4)
S1—C6—C10—C11	−7.0 (2)	N3—C18—C19—C22A	−172 (3)
N1—C1—C2—C3	−178.17 (15)	N3—C29—C30—C31	−177.72 (17)
N1—C1—C2—C5	5.7 (2)	N3—C29—C34—C33	177.01 (18)
N1—C12—C13—C14	−176.64 (17)	N4—C23—C27—C26	2.0 (3)
N1—C12—C17—C16	176.71 (17)	N4—C23—C27—C28	−170.91 (17)
N2—C6—C10—C9	−2.3 (3)	N4—C24—C25—C26	1.1 (3)
N2—C6—C10—C11	170.58 (17)	C18—S3—C23—N4	−155.97 (14)
N2—C7—C8—C9	−2.3 (4)	C18—S3—C23—C27	25.76 (16)
C1—S1—C6—N2	158.55 (14)	C18—N3—C28—O2	166.06 (17)
C1—S1—C6—C10	−23.70 (16)	C18—N3—C28—C27	−16.2 (2)
C1—N1—C11—O1	−161.38 (17)	C18—N3—C29—C30	131.40 (18)
C1—N1—C11—C10	21.7 (2)	C18—N3—C29—C34	−46.0 (2)
C1—N1—C12—C13	41.6 (2)	C18—C19—C20B—C21B	178.6 (6)
C1—N1—C12—C17	−135.66 (17)	C18—C19—C22B—S4B	−179.7 (3)
C1—C2—C3—C4	−177.30 (16)	C18—C19—C20A—C21A	−173 (3)
C1—C2—C5—S2	176.65 (13)	C18—C19—C22A—S4A	−174 (2)
C2—C3—C4—S2	0.9 (2)	C19—C20B—C21B—S4B	1.7 (11)
C3—C2—C5—S2	0.1 (2)	C19—C20A—C21A—S4A	−12 (7)
C4—S2—C5—C2	0.31 (15)	C20B—C19—C22B—S4B	0.0 (8)
C5—S2—C4—C3	−0.68 (16)	C21B—S4B—C22B—C19	0.8 (7)
C5—C2—C3—C4	−0.6 (2)	C22B—S4B—C21B—C20B	−1.5 (9)
C6—S1—C1—N1	52.23 (13)	C22B—C19—C20B—C21B	−1.1 (10)
C6—S1—C1—C2	−75.83 (12)	C23—S3—C18—N3	−53.18 (13)
C6—N2—C7—C8	1.6 (3)	C23—S3—C18—C19	74.24 (13)
C6—C10—C11—O1	−163.02 (18)	C23—N4—C24—C25	0.2 (3)
C6—C10—C11—N1	14.0 (3)	C23—C27—C28—O2	159.54 (18)
C7—N2—C6—S1	178.47 (15)	C23—C27—C28—N3	−18.3 (3)
C7—N2—C6—C10	0.8 (3)	C24—N4—C23—S3	179.98 (15)
C7—C8—C9—C10	0.6 (3)	C24—N4—C23—C27	−1.8 (3)
C8—C9—C10—C6	1.5 (3)	C24—C25—C26—C27	−0.8 (3)
C8—C9—C10—C11	−171.83 (19)	C25—C26—C27—C23	−0.6 (3)
C9—C10—C11—O1	9.8 (3)	C25—C26—C27—C28	172.74 (17)
C9—C10—C11—N1	−173.22 (17)	C26—C27—C28—O2	−13.3 (3)
C11—N1—C1—S1	−57.40 (18)	C26—C27—C28—N3	168.89 (16)
C11—N1—C1—C2	69.8 (2)	C28—N3—C18—S3	54.8 (2)
C11—N1—C12—C13	−130.96 (18)	C28—N3—C18—C19	−71.4 (2)
C11—N1—C12—C17	51.8 (2)	C28—N3—C29—C30	−53.0 (2)
C12—N1—C1—S1	129.98 (13)	C28—N3—C29—C34	129.62 (19)
C12—N1—C1—C2	−102.77 (17)	C29—N3—C18—S3	−129.72 (14)
C12—N1—C11—O1	11.0 (3)	C29—N3—C18—C19	104.08 (17)
C12—N1—C11—C10	−165.93 (15)	C29—N3—C28—O2	−9.4 (3)
C12—C13—C14—C15	−0.5 (3)	C29—N3—C28—C27	168.40 (15)
C13—C12—C17—C16	−0.6 (3)	C29—C30—C31—C32	0.6 (3)
C13—C14—C15—C16	0.4 (3)	C30—C29—C34—C33	−0.3 (3)
C14—C15—C16—C17	−0.3 (3)	C30—C31—C32—C33	0.0 (3)
C15—C16—C17—C12	0.4 (3)	C31—C32—C33—C34	−0.7 (3)
C17—C12—C13—C14	0.6 (3)	C32—C33—C34—C29	0.9 (3)
S3—C18—C19—C20B	53.2 (5)	C34—C29—C30—C31	−0.4 (3)
S3—C18—C19—C22B	−127.2 (5)	C20A—C19—C22A—S4A	10 (6)
S3—C18—C19—C20A	−124 (4)	C20A—C21A—S4A—C22A	15 (5)
S3—C18—C19—C22A	62 (3)	C21A—S4A—C22A—C19	−15 (5)
S3—C23—C27—C26	−179.89 (13)	C22A—C19—C20A—C21A	2 (7)
S3—C23—C27—C28	7.2 (2)		

### 3-Phenyl-2-(thiophen-3-yl)-2,3-dihydro-4H-pyrido[3,2-e][1,3]thiazin-4-one (1) Hydrogen-bond geometry (Å, º)

**Table:**

*D*—H···*A*	*D*—H	H···*A*	*D*···*A*	*D*—H···*A*
C4—H4···O1^i^	0.93	2.26	3.168 (2)	165
C18—H18···N2^ii^	0.98	2.53	3.494 (2)	167

Symmetry codes: (i) *x*−1, *y*, *z*; (ii) −*x*+1, −*y*+1, −*z*+1.

### 2-(1H-Indol-3-yl)-3-phenyl-2,3-dihydro-4H-pyrido[3,2-e][1,3]thiazin-4-one 0.438-hydrate (2) Crystal data

**Table:**

C_21_H_15_N_3_OS·0.438H_2_O	*F*(000) = 3032
*M**_r_* = 364.85	*D*_x_ = 1.168 Mg m^−^^3^
Monoclinic, *C*2/*c*	Cu *K*α radiation, λ = 1.54184 Å
*a* = 28.2424 (4) Å	Cell parameters from 15647 reflections
*b* = 11.0307 (1) Å	θ = 3.3–74.6°
*c* = 28.6936 (4) Å	µ = 1.51 mm^−^^1^
β = 111.952 (2)°	*T* = 173 K
*V* = 8290.9 (2) Å^3^	Block, clear colourless
*Z* = 16	0.2 × 0.2 × 0.17 mm

### 2-(1H-Indol-3-yl)-3-phenyl-2,3-dihydro-4H-pyrido[3,2-e][1,3]thiazin-4-one 0.438-hydrate (2) Data collection

**Table:**

ROD, Synergy Custom system, HyPix-Arc 150 diffractometer	8118 independent reflections
Radiation source: Rotating-anode X-ray tube, Rigaku (Cu) X-ray Source	6592 reflections with *I* > 2σ(*I*)
Mirror monochromator	*R*_int_ = 0.027
Detector resolution: 10.0000 pixels mm^-1^	θ_max_ = 76.1°, θ_min_ = 3.3°
ω scans	*h* = −34→35
Absorption correction: multi-scan (CrysAlisPro; Rigaku OD, 2023)	*k* = −13→11
*T*_min_ = 0.652, *T*_max_ = 1.000	*l* = −34→34
26357 measured reflections	

### 2-(1H-Indol-3-yl)-3-phenyl-2,3-dihydro-4H-pyrido[3,2-e][1,3]thiazin-4-one 0.438-hydrate (2) Refinement

**Table:**

Refinement on *F*^2^	Hydrogen site location: inferred from neighbouring sites
Least-squares matrix: full	H-atom parameters constrained
*R*[*F*^2^ > 2σ(*F*^2^)] = 0.068	*w* = 1/[σ^2^(*F*_o_^2^) + (0.1597*P*)^2^ + 0.3431*P*] where *P* = (*F*_o_^2^ + 2*F*_c_^2^)/3
*wR*(*F*^2^) = 0.218	(Δ/σ)_max_ = 0.001
*S* = 1.09	Δρ_max_ = 0.93 e Å^−^^3^
8118 reflections	Δρ_min_ = −0.25 e Å^−^^3^
492 parameters	Extinction correction: *SHELXL2018/3* (Sheldrick, 2015b), Fc^*^=kFc[1+0.001xFc^2^λ^3^/sin(2θ)]^-1/4^
560 restraints	Extinction coefficient: 0.00059 (7)
Primary atom site location: dual	

### 2-(1H-Indol-3-yl)-3-phenyl-2,3-dihydro-4H-pyrido[3,2-e][1,3]thiazin-4-one 0.438-hydrate (2) Special details

**Table:**

Geometry. All esds (except the esd in the dihedral angle between two l.s. planes) are estimated using the full covariance matrix. The cell esds are taken into account individually in the estimation of esds in distances, angles and torsion angles; correlations between esds in cell parameters are only used when they are defined by crystal symmetry. An approximate (isotropic) treatment of cell esds is used for estimating esds involving l.s. planes.

### 2-(1H-Indol-3-yl)-3-phenyl-2,3-dihydro-4H-pyrido[3,2-e][1,3]thiazin-4-one 0.438-hydrate (2) Fractional atomic coordinates and isotropic or equivalent isotropic displacement parameters (Å^2^)

**Table:**

	*x*	*y*	*z*	*U*_iso_*/*U*_eq_	Occ. (<1)
S1	0.26724 (2)	1.02257 (5)	0.46586 (2)	0.0607 (2)	
O1	0.29327 (7)	0.67587 (14)	0.40917 (7)	0.0681 (4)	
N1	0.30301 (7)	0.87957 (16)	0.40907 (7)	0.0550 (4)	
N2	0.18973 (8)	0.9065 (2)	0.47392 (8)	0.0682 (5)	
N3	0.17476 (8)	1.01209 (17)	0.28666 (7)	0.0651 (5)	
H3	0.154615	0.980900	0.258841	0.078*	
C1	0.28198 (8)	1.00078 (19)	0.40921 (8)	0.0526 (5)	
H1	0.308920	1.058872	0.411199	0.063*	
C2	0.27894 (9)	0.77749 (19)	0.41489 (8)	0.0551 (5)	
C3	0.23430 (8)	0.7943 (2)	0.43077 (8)	0.0570 (5)	
C4	0.22659 (8)	0.8966 (2)	0.45517 (8)	0.0563 (5)	
C5	0.15820 (11)	0.8124 (3)	0.46694 (12)	0.0790 (7)	
H5	0.132288	0.817730	0.479478	0.095*	
C6	0.16178 (11)	0.7077 (3)	0.44220 (12)	0.0819 (8)	
H6	0.138430	0.645049	0.437607	0.098*	
C7	0.20094 (10)	0.6979 (2)	0.42435 (10)	0.0699 (6)	
H7	0.204796	0.627491	0.408270	0.084*	
C8	0.34927 (8)	0.87271 (19)	0.39858 (8)	0.0540 (5)	
C9	0.34817 (11)	0.9027 (2)	0.35146 (9)	0.0679 (6)	
H9	0.317518	0.921538	0.325509	0.081*	
C10	0.39417 (13)	0.9043 (3)	0.34361 (11)	0.0783 (7)	
H10	0.394180	0.923974	0.312100	0.094*	
C11	0.43956 (11)	0.8768 (2)	0.38238 (12)	0.0758 (7)	
H11	0.470041	0.878957	0.377004	0.091*	
C12	0.43979 (11)	0.8469 (3)	0.42784 (12)	0.0799 (7)	
H12	0.470460	0.827601	0.453652	0.096*	
C13	0.39456 (9)	0.8446 (2)	0.43673 (10)	0.0670 (6)	
H13	0.395056	0.824079	0.468345	0.080*	
C14	0.23751 (8)	1.02915 (18)	0.36249 (8)	0.0514 (5)	
C15	0.21385 (9)	0.9531 (2)	0.32312 (8)	0.0587 (5)	
H15	0.222979	0.872738	0.321317	0.070*	
C16	0.17313 (10)	1.1292 (2)	0.30193 (9)	0.0612 (5)	
C17	0.14068 (13)	1.2233 (3)	0.27683 (11)	0.0810 (8)	
H17	0.114825	1.211520	0.245580	0.097*	
C18	0.14847 (15)	1.3332 (3)	0.30004 (13)	0.0939 (10)	
H18	0.127790	1.398271	0.284149	0.113*	
C19	0.18725 (15)	1.3503 (2)	0.34765 (12)	0.0860 (9)	
H19	0.191610	1.426651	0.362375	0.103*	
C20	0.21896 (11)	1.2571 (2)	0.37311 (9)	0.0649 (6)	
H20	0.244057	1.269320	0.404791	0.078*	
C21	0.21210 (9)	1.14314 (18)	0.34960 (8)	0.0533 (5)	
S2	0.53208 (2)	0.80892 (5)	0.57938 (2)	0.0617 (2)	
O2	0.60256 (8)	0.53259 (16)	0.68886 (7)	0.0789 (6)	
N4	0.61311 (8)	0.70766 (16)	0.65308 (6)	0.0579 (5)	
N5	0.46962 (7)	0.6310 (2)	0.53623 (8)	0.0701 (5)	
N6	0.66329 (8)	0.6096 (2)	0.53522 (9)	0.0724 (6)	
H6A	0.681683	0.551779	0.530967	0.087*	
C22	0.60106 (8)	0.78069 (19)	0.60738 (7)	0.0535 (5)	
H22	0.617659	0.859538	0.617745	0.064*	
C23	0.58928 (9)	0.6000 (2)	0.65250 (8)	0.0590 (5)	
C24	0.54443 (8)	0.5693 (2)	0.60636 (8)	0.0566 (5)	
C25	0.51395 (8)	0.6560 (2)	0.57346 (8)	0.0558 (5)	
C26	0.45593 (10)	0.5135 (3)	0.53047 (11)	0.0796 (8)	
H26	0.425467	0.493622	0.504472	0.095*	
C27	0.48337 (11)	0.4214 (3)	0.55987 (11)	0.0812 (8)	
H27	0.471970	0.341705	0.553716	0.097*	
C28	0.52867 (10)	0.4490 (2)	0.59919 (10)	0.0696 (6)	
H28	0.548071	0.388435	0.620286	0.084*	
C29	0.64864 (9)	0.7565 (2)	0.69909 (8)	0.0603 (6)	
C30	0.62963 (12)	0.7931 (2)	0.73587 (10)	0.0736 (7)	
H30	0.595434	0.781919	0.730879	0.088*	
C31	0.66289 (15)	0.8460 (3)	0.77949 (10)	0.0847 (8)	
H31	0.651188	0.868089	0.804590	0.102*	
C32	0.71196 (15)	0.8660 (3)	0.78611 (11)	0.0872 (9)	
H32	0.733482	0.904643	0.815103	0.105*	
C33	0.73048 (14)	0.8301 (3)	0.75058 (13)	0.0976 (10)	
H33	0.764686	0.842240	0.755844	0.117*	
C34	0.69784 (12)	0.7750 (3)	0.70623 (10)	0.0830 (8)	
H34	0.710222	0.751269	0.681804	0.100*	
C35	0.62190 (8)	0.7274 (2)	0.57069 (8)	0.0540 (5)	
C36	0.65085 (9)	0.6248 (2)	0.57640 (9)	0.0627 (6)	
H36	0.660523	0.573444	0.604098	0.075*	
C37	0.64177 (9)	0.7015 (3)	0.50155 (9)	0.0671 (6)	
C38	0.64240 (12)	0.7204 (3)	0.45374 (11)	0.0857 (8)	
H38	0.659817	0.668545	0.440134	0.103*	
C39	0.61610 (14)	0.8191 (4)	0.42777 (12)	0.0997 (10)	
H39	0.616231	0.834870	0.395984	0.120*	
C40	0.58901 (13)	0.8970 (3)	0.44741 (11)	0.0864 (8)	
H40	0.570973	0.962260	0.428514	0.104*	
C41	0.58924 (10)	0.8765 (2)	0.49478 (9)	0.0682 (6)	
H41	0.571843	0.928847	0.508220	0.082*	
C42	0.61552 (8)	0.7774 (2)	0.52264 (8)	0.0570 (5)	
O3	0.435729	0.497409	0.668991	0.203 (7)	0.25
O4	0.474029	0.693590	0.701120	0.222 (8)	0.25
O6	0.420529	0.560530	0.655540	0.175 (6)	0.25
O5	0.500000	0.050190	0.750000	0.231 (12)	0.25

### 2-(1H-Indol-3-yl)-3-phenyl-2,3-dihydro-4H-pyrido[3,2-e][1,3]thiazin-4-one 0.438-hydrate (2) Atomic displacement parameters (Å^2^)

**Table:**

	*U*^11^	*U*^22^	*U*^33^	*U*^12^	*U*^13^	*U*^23^
S1	0.0596 (4)	0.0634 (4)	0.0523 (3)	−0.0050 (2)	0.0130 (2)	−0.0096 (2)
O1	0.0792 (11)	0.0544 (9)	0.0689 (10)	−0.0008 (8)	0.0255 (9)	−0.0055 (7)
N1	0.0507 (10)	0.0517 (9)	0.0593 (10)	−0.0003 (7)	0.0169 (8)	0.0002 (8)
N2	0.0568 (11)	0.0810 (13)	0.0657 (11)	0.0064 (10)	0.0217 (9)	0.0143 (10)
N3	0.0691 (12)	0.0567 (10)	0.0541 (10)	−0.0052 (9)	0.0053 (9)	−0.0016 (8)
C1	0.0479 (11)	0.0505 (10)	0.0551 (11)	−0.0069 (8)	0.0145 (9)	−0.0044 (9)
C2	0.0559 (12)	0.0529 (11)	0.0485 (10)	−0.0029 (9)	0.0101 (9)	−0.0025 (9)
C3	0.0501 (11)	0.0606 (12)	0.0516 (11)	−0.0046 (9)	0.0089 (9)	0.0029 (9)
C4	0.0494 (11)	0.0639 (12)	0.0477 (10)	0.0010 (9)	0.0092 (8)	0.0060 (9)
C5	0.0585 (14)	0.0924 (19)	0.0853 (18)	0.0063 (13)	0.0260 (13)	0.0249 (15)
C6	0.0577 (14)	0.0852 (18)	0.0928 (19)	−0.0136 (13)	0.0165 (13)	0.0217 (15)
C7	0.0657 (14)	0.0644 (14)	0.0688 (14)	−0.0113 (11)	0.0129 (11)	0.0072 (11)
C8	0.0540 (11)	0.0495 (10)	0.0566 (11)	−0.0006 (9)	0.0183 (9)	−0.0026 (9)
C9	0.0726 (15)	0.0703 (14)	0.0599 (13)	0.0040 (12)	0.0238 (11)	−0.0010 (11)
C10	0.0999 (19)	0.0736 (15)	0.0756 (15)	−0.0018 (14)	0.0489 (14)	−0.0050 (13)
C11	0.0685 (15)	0.0715 (15)	0.0983 (18)	0.0020 (12)	0.0436 (14)	−0.0098 (13)
C12	0.0576 (14)	0.0897 (19)	0.0889 (18)	0.0108 (13)	0.0236 (13)	−0.0037 (15)
C13	0.0573 (13)	0.0782 (15)	0.0621 (13)	0.0048 (11)	0.0184 (10)	0.0021 (11)
C14	0.0523 (11)	0.0464 (10)	0.0516 (10)	−0.0042 (8)	0.0149 (9)	0.0013 (8)
C15	0.0634 (13)	0.0461 (10)	0.0577 (12)	−0.0026 (9)	0.0124 (10)	−0.0031 (9)
C16	0.0668 (14)	0.0544 (12)	0.0571 (12)	−0.0005 (10)	0.0168 (10)	0.0063 (9)
C17	0.091 (2)	0.0725 (16)	0.0681 (15)	0.0140 (14)	0.0166 (14)	0.0171 (12)
C18	0.126 (3)	0.0602 (15)	0.0856 (19)	0.0237 (17)	0.0287 (18)	0.0215 (14)
C19	0.130 (3)	0.0457 (12)	0.0895 (19)	0.0061 (14)	0.0501 (19)	0.0036 (12)
C20	0.0849 (17)	0.0483 (11)	0.0652 (13)	−0.0093 (11)	0.0322 (12)	−0.0015 (10)
C21	0.0590 (12)	0.0462 (10)	0.0560 (11)	−0.0059 (9)	0.0229 (9)	0.0012 (8)
S2	0.0612 (4)	0.0578 (3)	0.0657 (4)	0.0043 (2)	0.0234 (3)	0.0001 (2)
O2	0.0867 (13)	0.0657 (10)	0.0587 (9)	−0.0204 (9)	−0.0021 (9)	0.0080 (8)
N4	0.0648 (11)	0.0555 (10)	0.0444 (8)	−0.0153 (8)	0.0100 (8)	−0.0063 (7)
N5	0.0468 (10)	0.0849 (14)	0.0693 (12)	−0.0012 (9)	0.0111 (9)	−0.0054 (10)
N6	0.0487 (10)	0.0872 (14)	0.0779 (13)	−0.0076 (10)	0.0200 (9)	−0.0315 (11)
C22	0.0580 (12)	0.0528 (11)	0.0454 (10)	−0.0100 (9)	0.0144 (9)	−0.0081 (8)
C23	0.0604 (13)	0.0559 (11)	0.0509 (11)	−0.0120 (10)	0.0093 (9)	−0.0062 (9)
C24	0.0514 (11)	0.0574 (11)	0.0533 (11)	−0.0089 (9)	0.0108 (9)	−0.0079 (9)
C25	0.0470 (11)	0.0649 (12)	0.0531 (11)	−0.0014 (9)	0.0157 (9)	−0.0067 (9)
C26	0.0504 (13)	0.0942 (19)	0.0770 (17)	−0.0174 (13)	0.0041 (12)	−0.0155 (14)
C27	0.0620 (15)	0.0706 (15)	0.0933 (19)	−0.0201 (13)	0.0088 (13)	−0.0215 (14)
C28	0.0621 (14)	0.0581 (12)	0.0735 (15)	−0.0118 (11)	0.0078 (11)	−0.0079 (11)
C29	0.0691 (14)	0.0546 (12)	0.0462 (10)	−0.0156 (10)	0.0087 (9)	−0.0064 (9)
C30	0.0821 (17)	0.0706 (15)	0.0602 (13)	0.0044 (13)	0.0175 (12)	−0.0113 (11)
C31	0.116 (2)	0.0689 (16)	0.0579 (14)	0.0003 (16)	0.0200 (15)	−0.0152 (12)
C32	0.115 (2)	0.0640 (15)	0.0599 (14)	−0.0278 (15)	0.0061 (15)	−0.0065 (12)
C33	0.084 (2)	0.114 (3)	0.0811 (19)	−0.0478 (19)	0.0149 (15)	−0.0072 (17)
C34	0.0751 (17)	0.111 (2)	0.0605 (14)	−0.0343 (16)	0.0224 (12)	−0.0107 (14)
C35	0.0450 (10)	0.0602 (12)	0.0512 (10)	−0.0135 (9)	0.0116 (8)	−0.0146 (9)
C36	0.0482 (11)	0.0699 (14)	0.0628 (12)	−0.0092 (10)	0.0123 (10)	−0.0151 (10)
C37	0.0498 (12)	0.0871 (16)	0.0625 (13)	−0.0246 (11)	0.0190 (10)	−0.0252 (12)
C38	0.0764 (17)	0.118 (2)	0.0719 (15)	−0.0254 (16)	0.0384 (14)	−0.0273 (15)
C39	0.097 (2)	0.139 (3)	0.0708 (16)	−0.0375 (19)	0.0406 (16)	−0.0105 (17)
C40	0.091 (2)	0.097 (2)	0.0706 (16)	−0.0297 (16)	0.0293 (15)	0.0030 (15)
C41	0.0668 (14)	0.0730 (15)	0.0626 (13)	−0.0222 (12)	0.0217 (11)	−0.0049 (11)
C42	0.0508 (11)	0.0656 (12)	0.0523 (10)	−0.0211 (10)	0.0165 (9)	−0.0158 (9)
O3	0.205 (10)	0.219 (11)	0.213 (11)	−0.027 (8)	0.108 (8)	0.011 (8)
O4	0.231 (12)	0.216 (11)	0.203 (11)	0.018 (9)	0.062 (9)	0.024 (8)
O6	0.181 (9)	0.184 (9)	0.166 (9)	0.024 (8)	0.071 (7)	0.047 (7)
O5	0.211 (15)	0.237 (15)	0.240 (15)	0.000	0.081 (10)	0.000

### 2-(1H-Indol-3-yl)-3-phenyl-2,3-dihydro-4H-pyrido[3,2-e][1,3]thiazin-4-one 0.438-hydrate (2) Geometric parameters (Å, º)

**Table:**

S1—C1	1.838 (2)	S2—C22	1.835 (2)
S1—C4	1.755 (2)	S2—C25	1.753 (2)
O1—C2	1.223 (3)	O2—C23	1.220 (3)
N1—C1	1.464 (3)	N4—C22	1.467 (3)
N1—C2	1.357 (3)	N4—C23	1.362 (3)
N1—C8	1.448 (3)	N4—C29	1.432 (3)
N2—C4	1.343 (3)	N5—C25	1.336 (3)
N2—C5	1.333 (4)	N5—C26	1.345 (4)
N3—C15	1.369 (3)	N6—C36	1.363 (3)
N3—C16	1.371 (3)	N6—C37	1.375 (4)
C1—C14	1.488 (3)	C22—C35	1.504 (3)
C2—C3	1.504 (3)	C23—C24	1.489 (3)
C3—C4	1.387 (3)	C24—C25	1.392 (3)
C3—C7	1.387 (3)	C24—C28	1.391 (3)
C5—C6	1.379 (5)	C26—C27	1.362 (4)
C6—C7	1.386 (4)	C27—C28	1.387 (3)
C8—C9	1.381 (3)	C29—C30	1.410 (4)
C8—C13	1.372 (3)	C29—C34	1.342 (4)
C9—C10	1.399 (4)	C30—C31	1.382 (4)
C10—C11	1.381 (4)	C31—C32	1.345 (5)
C11—C12	1.343 (4)	C32—C33	1.367 (5)
C12—C13	1.393 (4)	C33—C34	1.401 (4)
C14—C15	1.365 (3)	C35—C36	1.370 (3)
C14—C21	1.426 (3)	C35—C42	1.432 (3)
C16—C17	1.394 (3)	C37—C38	1.394 (4)
C16—C21	1.407 (3)	C37—C42	1.397 (3)
C17—C18	1.361 (4)	C38—C39	1.370 (5)
C18—C19	1.408 (5)	C39—C40	1.402 (5)
C19—C20	1.379 (4)	C40—C41	1.375 (4)
C20—C21	1.406 (3)	C41—C42	1.394 (4)
			
C4—S1—C1	95.25 (10)	C25—S2—C22	95.91 (10)
C2—N1—C1	122.35 (18)	C23—N4—C22	121.32 (17)
C2—N1—C8	120.96 (17)	C23—N4—C29	120.80 (18)
C8—N1—C1	116.54 (16)	C29—N4—C22	117.79 (17)
C5—N2—C4	116.8 (2)	C25—N5—C26	116.0 (2)
C15—N3—C16	108.53 (19)	C36—N6—C37	109.2 (2)
N1—C1—S1	110.37 (14)	N4—C22—S2	109.75 (15)
N1—C1—C14	113.36 (17)	N4—C22—C35	112.98 (19)
C14—C1—S1	112.06 (15)	C35—C22—S2	112.90 (14)
O1—C2—N1	122.5 (2)	O2—C23—N4	121.9 (2)
O1—C2—C3	120.7 (2)	O2—C23—C24	120.6 (2)
N1—C2—C3	116.73 (19)	N4—C23—C24	117.4 (2)
C4—C3—C2	124.1 (2)	C25—C24—C23	123.5 (2)
C7—C3—C2	118.0 (2)	C28—C24—C23	117.8 (2)
C7—C3—C4	117.6 (2)	C28—C24—C25	118.2 (2)
N2—C4—S1	114.87 (18)	N5—C25—S2	115.41 (18)
N2—C4—C3	124.0 (2)	N5—C25—C24	123.9 (2)
C3—C4—S1	121.08 (17)	C24—C25—S2	120.72 (16)
N2—C5—C6	123.8 (3)	N5—C26—C27	124.9 (2)
C5—C6—C7	118.6 (3)	C26—C27—C28	118.6 (3)
C6—C7—C3	119.1 (3)	C27—C28—C24	118.5 (2)
C9—C8—N1	119.9 (2)	C30—C29—N4	117.8 (2)
C13—C8—N1	119.4 (2)	C34—C29—N4	121.8 (2)
C13—C8—C9	120.5 (2)	C34—C29—C30	120.3 (2)
C8—C9—C10	118.6 (2)	C31—C30—C29	118.6 (3)
C11—C10—C9	120.4 (3)	C32—C31—C30	120.8 (3)
C12—C11—C10	120.2 (3)	C31—C32—C33	120.6 (3)
C11—C12—C13	120.7 (3)	C32—C33—C34	119.9 (3)
C8—C13—C12	119.6 (2)	C29—C34—C33	119.8 (3)
C15—C14—C1	127.15 (19)	C36—C35—C22	127.7 (2)
C15—C14—C21	106.50 (19)	C36—C35—C42	106.7 (2)
C21—C14—C1	126.33 (19)	C42—C35—C22	125.6 (2)
C14—C15—N3	110.2 (2)	N6—C36—C35	109.5 (2)
N3—C16—C17	129.1 (2)	N6—C37—C38	129.5 (3)
N3—C16—C21	107.8 (2)	N6—C37—C42	107.7 (2)
C17—C16—C21	123.0 (2)	C38—C37—C42	122.8 (3)
C18—C17—C16	117.1 (3)	C39—C38—C37	116.7 (3)
C17—C18—C19	121.2 (3)	C38—C39—C40	122.4 (3)
C20—C19—C18	122.0 (3)	C41—C40—C39	119.7 (3)
C19—C20—C21	117.8 (3)	C40—C41—C42	120.0 (3)
C16—C21—C14	106.90 (19)	C37—C42—C35	106.9 (2)
C20—C21—C14	134.3 (2)	C41—C42—C35	134.6 (2)
C20—C21—C16	118.8 (2)	C41—C42—C37	118.5 (2)

### 2-(1H-Indol-3-yl)-3-phenyl-2,3-dihydro-4H-pyrido[3,2-e][1,3]thiazin-4-one 0.438-hydrate (2) Hydrogen-bond geometry (Å, º)

**Table:**

*D*—H···*A*	*D*—H	H···*A*	*D*···*A*	*D*—H···*A*
N3—H3···O2^i^	0.86	2.00	2.828 (3)	161
C26—H26···N6^ii^	0.93	2.60	3.463 (3)	155

Symmetry codes: (i) *x*−1/2, −*y*+3/2, *z*−1/2; (ii) −*x*+1, −*y*+1, −*z*+1.
